# Identification of Incidental Skin Cancers Among Adults Referred to Dermatologists for Suspicious Skin Lesions

**DOI:** 10.1001/jamanetworkopen.2020.30107

**Published:** 2020-12-16

**Authors:** Sharif Omara, David Wen, Benjamin Ng, Rakesh Anand, Rubeta N. Matin, Kathy Taghipour, Ben Esdaile

**Affiliations:** 1 Department of Cardiology, Leiden University Medical Centre, Albinusdreef, Leiden, the Netherlands; 2 Department of Dermatology, Oxford University Hospitals National Health Service Foundation Trust, Churchill Hospital, Headington, Oxford, United Kingdom; 3 Department of Dermatology, Whittington Health National Health Service Foundation Trust, London, United Kingdom

## Abstract

**Importance:**

The incidence of skin cancer is increasing and evaluation of the utility of total body skin examination (TBSE) in detecting incidental skin cancers is warranted.

**Objectives:**

To evaluate the proportion and rate of incidental skin cancer detection in urgent skin cancer clinics and investigate the rate of incidental skin cancer detection in 2 groups based on the degree of clinical suspicion of the index lesion for malignancy.

**Design, Setting, and Participants:**

A multicenter retrospective cohort study with a case note review of consecutive secondary care consultations was conducted using data from 2 urgent suspected skin cancer screening clinics in UK National Health Service trusts. The study was performed from January 1, 2015, to March 31, 2016, and data analysis was performed from October 14, 2018, to February 1, 2019. Patients included those presenting with a skin lesion suspicious of malignancy who were referred to the urgent suspected skin cancer clinic (N = 5944) over 15 months. Patients who accepted and received a TBSE were subsequently included in the analysis.

**Main Outcomes and Measures:**

The proportion and rate of incidental skin cancer detection through TBSE and whether a clinically suspicious (malignant) index lesion was associated with a higher chance of having a malignant incidental lesion.

**Results:**

Of the 5944 patients referred to the clinic, 4726 individuals (79.5%) were evaluated. In the cohort included in the analyses, the median age was 57 years (interquartile range, 39-73 years); 2567 patients (54.3%) were women. A total of 1117 skin cancers were identified; of these, 242 lesions (21.7%) were detected incidentally through TBSE, including 197 of 570 (34.6%) basal cell carcinomas, 16 of 250 (6.4%) squamous cell carcinomas, and 25 of 215 (11.6%) melanomas. The detection rate of incidental malignant lesions was 5.1 lesions per 100 patients examined (5.1%; 95% CI, 4.5%-5.8%). There was a higher detection rate of histologically confirmed incidental malignant lesions in individuals with clinically suspicious index lesions requiring biopsy (10.9%; 95% CI, 9.5%-12.5%) compared with those presenting with clinically benign index lesions (2.0%; 95% CI, 1.6%-2.5%) (*P* < .001).

**Conclusions and Relevance:**

The findings of this study support the use of TBSE for urgent skin cancer referrals, highlighting the potential harms of solitary lesion assessment in a subgroup. Individuals presenting with a clinically suspicious index lesion requiring biopsy are most likely to benefit from TBSE and should be counseled regarding the benefit.

## Key Points

**Question:**

What is the rate of incidental skin cancer detection in urgent skin cancer clinics, and are incidental cancers more likely to be detected in patients with a clinically suspicious index lesion than in those without?

**Findings:**

In a cohort study including 4726 patients, 1117 malignant lesions were detected, 22% of which were identified incidentally by total body skin examinations corresponding to an incidental lesion detection rate of 5.1%. Detection of a malignant incidental lesion by total body skin examinations was significantly more likely in patients presenting with an index lesion suspicious for malignancy, compared with patients who presented with index lesions judged to be clinically benign.

**Meaning:**

The findings of this study suggest that total body skin examinations may be useful for detecting incidental skin cancers and that patients with suspicious index lesions should be prioritized.

## Teaser

This cohort study examines the use of total body skin examinations for detection of incidental skin cancers in patients referred to dermatology clinics for evaluation of a clinically suspicious index lesion.

## Introduction

The incidence of skin cancers is rapidly increasing in the UK, with corresponding increases in mortality, morbidity, and health care costs.^1,2,3,4,5,6,7^ This increase appears to be primarily influenced by an aging population, increased recreational sun exposure, and tanning bed use leading to higher lifetime UV exposure in conjunction with possible earlier detection.^8,9^

In the UK National Health Service, patients with suspected malignant melanoma (MM) or squamous cell carcinoma (SCC) are referred from general practitioners (primary care) to secondary care dermatology services using an urgent suspected skin cancer referral pathway within 2 weeks.^10^ Patients with suspected basal cell carcinomas (BCCs) are also referred under this pathway if delays could affect the patient owing to factors such as the size or site of the lesion.^10^

Total body skin examination (TBSE) is the systematic examination of a patient’s entire skin surface, nails, hair, and relevant mucosal surfaces aiming to identify incidental skin cancers that the patient may not be able to see, leading to earlier cancer detection.^11,12,13^ Screening of the general population using TBSE remains controversial,^14^ with ambiguity about which sites should be included in TBSE^15^ and concerns about cost-effectiveness.^16^ In 1 UK study, as many as a third of melanomas were incidental lesions detected by TBSE,^11^ with higher proportions reported in other countries where UV exposure may be greater.^12,13,17^ Based on these data, Oxford University Hospitals and Whittington Hospital Health National Health Service Foundation trusts offer TBSE to all new patients referred to our urgent skin cancer screening clinics to maximize yield in detecting incidental lesions.

Nevertheless, routine use of TBSE by dermatologists is variable; in a survey of 464 dermatologists, 30% reported performing TBSE on all patients, but 49% reported performing TBSE only on high-risk patients.^18^ The UK National Institute for Health and Care Excellence guidelines propose that all patients with pigmented skin lesions referred to secondary or tertiary care should be assessed using dermoscopy,^19^ and performing TBSE in conjunction with dermoscopy in a skin cancer clinic takes an average of 2 to 6 minutes to complete.^16,20^ Within the time constraints of a 10- to 15-minute outpatient consultation, it is not surprising that 42% of dermatologists lacked sufficient time to perform TBSE.^18^ In addition, a survey of 251 patients reported that 8% felt embarrassed to undergo TBSE and 4% did not want a dermatologist to regularly perform TBSE.^21^ In addition, 44.2% of dermatologists reported that patient embarrassment was a major or moderate barrier to performing TBSE in skin cancer screening.^22^ Thus, identifying a specific patient subgroup to target for TBSE could optimize detection rates of incidental cancers and ensure that skin cancer screening clinics perform efficiently.

This study aimed to evaluate the rate and proportion of incidental skin cancer detection by TBSE in 2 tertiary referral centers. We also investigated whether the rate of incidental skin cancer detection was greater in patients who presented with a clinically suspicious index lesion requiring biopsy (group A) compared with patients who presented with clinically benign index lesions (group B).

## Methods

A retrospective cohort study with case-note review was undertaken in 2 UK National Health Service dermatology centers (Whittington Hospital, London, and Churchill Hospital, Oxford). Approval for collection of these retrospective anonymized data was obtained from local National Health Service Trust Research & Development departments that did not require formal human ethics review. This study followed the Strengthening the Reporting of Observational Studies in Epidemiology (STROBE) reporting guideline for cohort studies.

Both centers have dedicated urgent skin cancer screening clinics providing rapid access for referrals from primary care. Both centers use specific clinic proformas to ensure that standardized demographic information and clinical findings are recorded, including any planned surgical intervention following the consultation. The proforma also contains a skin check tick box where the practitioner can record whether full or partial TBSE was performed or declined. All urgent skin cancer screening clinics are consultant led and all lesions are reviewed by a consultant dermatologist before surgical excision. All dermatologists working in these clinics were specifically trained in dermoscopy and all lesions suspicious for skin cancer that were biopsied were dual reported by 2 specialist-accredited consultant dermatopathologists.

All clinic proformas for patients attending the urgent skin cancer screening clinic during a period of 15 months (January 1, 2015, to March 31, 2016) were reviewed. Patients who declined TBSE, had partial skin examination, did not have any documentation of TBSE being performed, or had incomplete records were excluded from analyses (n = 1218). Data analysis was performed from October 14, 2018, to February 1, 2019.

An index lesion was defined as any lesion identified by the general practitioner before the consultation that was the subject of referral or a lesion brought to the dermatologist’s attention by the patient before the patient was examined. An incidental lesion was any new suspicious skin lesion identified during TBSE during the consultation. Skin cancer (malignant) lesions included BCC, SCC, MM, lentigo maligna melanoma (LMM), melanoma in situ (MIS), lentigo maligna (LM), keratoacanthoma, keratoacanthomalike SCCs, and other malignant lesions, which included mycosis fungoides, Merkel cell carcinoma, sarcoma, and cutaneous lymphoproliferative disorders. Premalignant lesions included severely dysplastic nevi, actinic keratoses, and Bowen disease.

**Figure 1.**
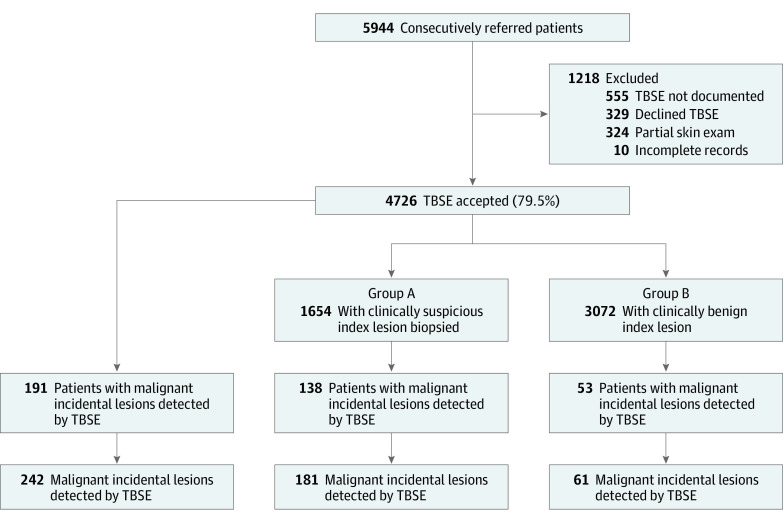
Number of Patients Accepting Total Body Skin Examination (TBSE) and the Number of Malignant Incidental Lesions Detected by Group

Individuals included in the study cohort were divided into 2 groups (Figure 1). Group A included individuals who presented with an index lesion clinically suspicious for premalignancy or malignancy warranting biopsy. Group B included individuals who presented with an index lesion that was judged to be clinically benign and was managed conservatively (including no treatment) or rarely removed for symptomatic relief.

Overall, 66 patients had more than 1 incidental lesion biopsied. For the statistical analyses comparing patients with malignant lesions in groups A and B, the unit of analysis used was the patient. In the case of 2 or more lesions in 1 patient, the highest-grade lesion was used in the analysis.

### Statistical Analysis

The rate of lesion detection was calculated using the total number of lesions identified per patient examined. SPSS, version 25 (IBM Corp) was used for statistical analyses. Rates and proportions expressed as 95% CIs were calculated using the Wilson score interval. A Mann-Whitney test was used in the analysis of age; χ^2^ tests were used in all other analyses. Tests were 2-sided and unpaired, and the significance threshold was set at *P* < .05.

## Results

Of 5944 patients referred to an urgent skin cancer screening clinic, TBSE was accepted by 4726 individuals (79.5%), partially completed for 324 patients (5.5%), and declined by 329 patients (5.5%); 555 patients (9.3%) did not have documentation of whether TBSE was performed. In the cohort of 4726 patients who accepted TBSE, 2567 individuals were women (54.3%) and 2159 individuals (45.7%) were men; median age was 57 years (interquartile range [IQR], 39-73 years). A total of 1654 patients (35.0%) presented with a clinically suspicious index lesion requiring biopsy (group A) and 3072 patients (65.0%) presented with benign index lesions (group B) (Figure 1).

For the entire cohort, 242 of 1117 malignant lesions (21.7%) were detected incidentally, corresponding to a rate of 5.1% (5.1 incidental lesions detected per 100 patients receiving TBSE; 95% CI, 4.5%-5.8%). The breakdown per lesion type confirmed by histologic examination is displayed in Table 1. Of the 242 malignant lesions detected incidentally, 197 lesions (81.4%) were BCCs, followed by melanomas (25 of 215 [11.6%]; 12 MM/LMM and 13 MIS/LM), SCCs (16 [6.4%]), and keratoacanthomas (2 [3.3%]). The 2 other incidentally detected cancers were mycosis fungoides and synovial sarcoma. Seventy-eight of 197 BCCs (39.6%) detected incidentally were located on the face and 12 BCCs (6.1%) were located on the neck. The location of all malignant incidental lesions by patient’s sex is summarized in the eFigure in the Supplement.

**Table 1.** Histopathologic Data for Lesions Identified

Histopathologic diagnosis	Index lesion, No.	Incidental lesion, No.	Total, No.	Proportion of lesions identified incidentally, %
Basal cell carcinoma	373	197	570	34.6
Squamous cell carcinoma	234	16	250	6.4
Malignant melanoma/lentigo maligna melanoma	109	12	121	9.9
Melanoma in situ/lentigo maligna	81	13	94	13.8
Keratoacanthoma	58	2	60	3.3
Other malignant lesion	20	2	22	9.1
Actinic keratosis	83	28	111	25.2
Bowen disease	69	25	94	26.6
Severely dysplastic nevus	49	8	57	14.0
Total				
Premalignant and malignant lesions	1076	303	1379	22.0
Malignant lesions	875	242	1117	21.7

The age distribution of patients with index and incidental melanomas (malignant and in situ) is summarized in eTable 1 in the Supplement. The median age of the patients with index melanomas was 64 years (IQR, 52-73) years. By definition, all index melanomas were in the group A cohort. Median age of detection for patients with incidental melanomas was 78 years (IQR, 60-84 years).

The 25 incidental melanomas detected were in 23 patients, with 1 patient having 3 incidental malignant melanomas. The Breslow thickness for melanomas is summarized in eTable 2 in the Supplement.

### Detection Rates per Group

The detection rates of incidental malignant and premalignant lesions identified by TBSE for the entire cohort and per group are summarized in Table 2. Use of TBSEs to screen resulted in higher rates of skin cancer detection compared with lesion-directed screening and general population screening. Of 1654 patients in group A, 138 patients (8.3%) had 1 or more malignant lesions detected incidentally on TBSE. A total of 181 malignant lesions were detected in group A, corresponding to a detection rate of 10.9% (95% CI, 9.5%-12.5%). Fifty-three of 3072 patients (1.7%) in group B had 1 or more malignant lesions detected incidentally on TBSE. A total of 61 malignant lesions were detected in group B, corresponding to a detection rate of 2.0% (95% CI, 1.6%-2.5%) The rate of malignant incidental lesion detection was significantly greater in group A compared with group B (*P* < .001).

**Table 2.** Patient Characteristics and Rates of Incidental Lesion Detection

Variable	Patients accepting TBSE	Group A	Group B	*P* value^a^
No. of patients (%)	4726	1654 (35.0)	3072 (65.0)	NA
Age, median (IQR), y	57 (39-73)	67 (48-79)	52 (36-68)	<.001
Sex, No. (%)				
Women	2567 (54.3)	760 (45.9)	1807 (58.8)	<.001
Men	2159 (45.7)	894 (54.1)	1265 (41.2)	<.001
Patients with incidental malignant or premalignant lesions detected on TBSE, No. (%)	236 (5.0)	174 (10.5)	62 (2.0)	<.001
Patients with incidental malignant lesions detected on TBSE, No.(%)	191 (4.0)	138 (8.3)	53 (1.7)	<.001
Incidental malignant or premalignant lesions, No.	303	229	74	
Rate of detection, % (95% CI)	6.4 (5.8-7.2)	13.8 (12.3-15.6)	2.4 (1.9-3.0)	<.001
Incidental malignant lesions, No.	242	181	61	
Rate of detection, % (95% CI)	5.1 (4.5-5.8)	10.9 (9.5-12.5)	2.0 (1.6-2.5)	<.001
Malignant or in situ melanomas, No.	25	17	8	
Rate of detection, % (95% CI)	0.53 (0.36-0.78)	1.0 (0.64-1.6)	0.26 (0.13-0.51)	.002
Malignant melanomas, No.	12	6	6	
Rate of detection, % (95% CI)	0.25 (0.15-0.44)	0.36 (0.17-0.79)	0.20 (0.090-0.43)	.30
In situ melanomas, No.	13	11	2	
Rate of detection, % (95% CI)	0.28 (0.16-0.47)	0.67 (0.37-1.19)	0.07 (0.018-0.24)	<.001

Abbreviations: IQR, interquartile range; NA, not applicable; TBSE, total body skin examination.

^a^ A Mann-Whitney test was used in the analysis of age; χ^2^ tests were used in all other analyses.

**Figure 2.**
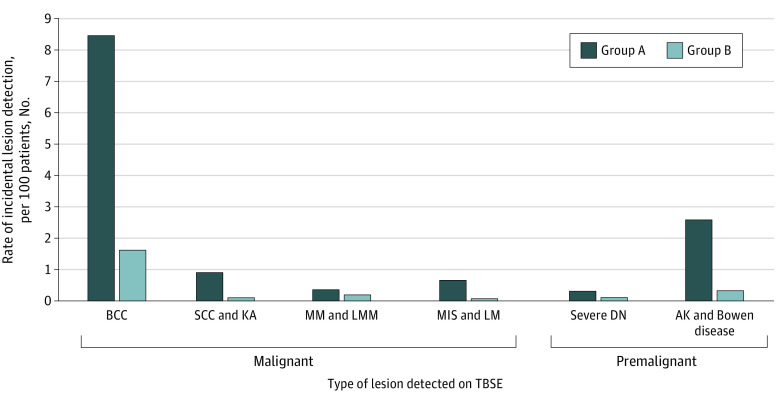
Rates of Detection of Incidental Skin Lesions by Total Body Skin Examination (TBSE) According to Skin Lesion Type Abbreviations: AK, actinic keratosis; BCC, basal cell carcinoma; DN, severely dysplastic nevus; KA, keratoacanthoma; LM, lentigo maligna; LMM, lentigo maligna melanoma; MIS, melanoma in situ; MM, malignant melanoma; SCC, squamous cell carcinoma.

Considering malignant and premalignant lesions together, the rate of detection of incidental lesions was also significantly greater in group A (13.8%; 95% CI, 12.3%-15.6%) compared with group B (2.4%; 95% CI, 1.9%-3.0%) (*P* < .001). In group A, fewer patients needed to be examined to detect 1 incidental malignant lesion. Categorizing patients into the 2 groups showed an older and predominantly male cohort in group A. Rates of detection for all types of incidental malignant or premalignant lesions were greater in group A compared with group B (Figure 2).

Overall, 25 melanomas were detected incidentally (12 MMs and 13 MISs): 17 in group A and 8 in group B, corresponding to an overall detection rate of 0.53% (95% CI, 0.36%-0.78%) (Table 2). Detection rates were 1.0% (95% CI, 0.64%-1.6%) in group A and 0.26% (95% CI, 0.13%-0.51%) in group B (*P* = .002). However, when identifying MMs alone, 6 were detected in each group (*P* = .30).

### Detection of Multiple Incidental Lesions

Biopsies were performed on more than 1 incidental lesion in 66 patients. Table 3 reports the number of incidental lesions biopsied for a single patient (maximum, 5). Forty-nine patients had more than 1 incidental lesion biopsied in group A, compared with 17 patients from group B. Histopathologic diagnoses of incidental lesions included 106 malignant lesions, 19 premalignant lesions, and 35 benign lesions.

**Table 3.** Number of Patients Who Underwent Multiple Biopsies for Suspicious Incidental Lesions

Variable	No. (%)		Total
	Group A	Group B	
Incidental lesion biopsies, No.			
2	33 (73.3)	12 (26.7)	45
3	12 (75.0)	4 (25.0)	16
4	2 (66.7)	1 (33.3)	3
5	2 (100)	0	2
Total	49 (74.2)	17 (25.8)	66
Incidental lesion, type			
Basal cell carcinoma	75 (84.3)	14 (15.7)	89
Squamous cell carcinoma	7 (100)	0	7
Malignant melanoma	1 (20.0)	4 (80.0)	5
Keratoacanthoma	1 (100)	0	1
Actinic keratosis	2 (33.3)	4 (66.7)	6
Bowen disease	11 (91.7)	1 (8.3)	12
Benign	18 (51.4)	17 (48.6)	35
Severely dysplastic nevus	1 (100)	0	1
Melanoma in situ	4 (100)	0	4
Total	120 (75.0)	40 (25.0)	160

## Discussion

This retrospective, multicenter cohort study noted that, of 1117 skin cancers identified in urgently referred patients, 242 lesions (21.7%) were detected incidentally through TBSE, with a detection rate of 5.1 incidental lesions per 100 patients examined (5.1%). These lesions were mainly BCCs (197 of 570 [34.6%]), followed by SCCs (16 of 250 [6.4%]) and melanomas (MM/LMM or MIS/LM, 25 of 215 [11.6%]).

In comparison with these findings, in a UK study involving 2 Scottish hospitals, the index melanoma detection rate was 1.3% (24 of 1851 patients), and the incidental melanoma detection rate was 0.76% (14 of 1851 patients).^11^ The rate of malignant incidental lesion detection in 1 of these hospitals was 4.76%. The proportion of incidental SCCs in our study (6.4%) was comparable to that reported in a similar retrospective review conducted at a tertiary center in the UK, where the proportion of SCCs detected incidentally was 6.8% (32 of 469).^23^

Our study used TBSEs to screen for incidental malignant lesions in patients referred urgently to a secondary care dermatology clinic. In a Belgian study of lesion-directed screening, skin examination was offered to patients who self-identified lesions meeting certain criteria, such as a new lesion present for longer than 4 weeks or having 1 of the ABCD (asymmetry, border irregularity, color that is not uniform, diameter >6 mm, and evolving size, shape, or color) properties^16^; 248 of 9484 individuals (3.2%) self-presented for lesion-directed screening that detected 8 malignant lesions. In the same study, a separate, comparable population was offered TBSE and 1668 of 9325 participants accepted, leading to 39 incidental malignant lesions detected, corresponding to a detection rate of 2.3%. To our knowledge, the SCREEN project in northern Germany is the largest European study to date investigating TBSE in general population screening; 3103 malignant skin tumors were detected from 360 288 TBSEs in a population of 1.88 million, giving a detection rate of 0.86%.^24^ Performing TBSEs in our patient subgroup resulted in a higher yield than in general population screening and lesion-directed screening.

A key finding from our study is that dermatologists were more likely to detect incidental premalignant or malignant lesions if patients presented with a clinically suspicious (potentially malignant) lesion requiring biopsy. The detection rate of malignant lesions in group A was 10.9% and the number needed to examine to detect a malignant or premalignant lesion is 9 patients. Compared with use of TBSEs in patients in group B, dermatologists were more than 5 times more likely to detect an incidental malignant lesion with use of TBSE in patients in group A. Detection rates were higher for all types of malignant or premalignant skin tumors in group A, including invasive and in situ melanoma (Figure 2). However, the rate of detection of MMs was not significantly higher in group A. Three of the 6 MMs in group B were in a single patient, whereas the 9 other MMs were found in 9 other patients. Owing to the small number of incidental MMs detected in each group, this very high-risk patient may have positively skewed the rate of MM detection in group B.

A group of 66 patients had biopsies of multiple incidental lesions performed, further supporting the utility of TBSE in patients referred to an urgent skin cancer clinic, because, for some patients, multiple skin cancers (up to 5) were detected that were distinct from the lesion indicated in the initial referral. Patients who had 2 or more incidental lesions biopsied were more commonly present in group A (49 patients) than group B (17 patients), further supporting our observation that group A was at high risk for multiple lesions occurring contemporaneously.

Even though the detection rate of incidental malignant lesions for patients in group B was lower (2.0%), incidental skin cancers were still detected, including invasive and in situ melanomas. Thus, based on our data, we recommend offering TBSEs for all patients who are urgently referred to a tumor clinic (groups A and B) if resources allow. However, if time constraints exist, we recommend TBSE to be prioritized in individuals who have a clinically suspicious index skin lesion (group A).

### Teledermatology

Teledermatology (with potential teledermoscopy) can reduce in-person dermatology clinic visits by up to 61.5%, and clinical outcomes have been comparable with conventional care; only diagnostic accuracy is possibly inferior to in-person examinations, with interobserver agreement percentage ranging from 41% to 100%.^25,26^ Saving travel time and accessibility are often cited as positive features.^27,28,29^ As a result of the coronavirus disease 2019 pandemic, teledermatology has been increasingly used to manage all skin conditions to reduce face-to-face consultations^30^ and has been shown to have an important educational role^31^ while improving quality of life.^32,33^ A lengthy waiting time before dermatologist consultation is a risk factor for pigmented skin lesions; although teledermatology can reduce waiting times (from 88.6 to 12.3 days), there is evidence suggesting that results are suboptimal for diagnosing such lesions.^34,35^

Teledermatology was not part of the patient pathway that we examined in this study; solitary examination of a lesion in this setting would have missed 21.7% of malignant lesions (predominately BCCs) detected incidentally as well as premalignant lesions, including dysplastic nevi and Bowen disease. Total body skin examination and teledermatology do not currently appear to be compatible. In some models of teledermatology, patients are scheduled directly for skin surgery without TBSE.^36,37,38^ Our data suggest that patients with a clinically suspicious index lesion warranting biopsy (group A) would not be suitable for teledermatology, because these patients are most likely to have incidental skin cancers, which would be missed if teledermatology alone is used. Alternatively, if teledermatology identifies a clinically suspicious index lesion, we recommend that patients should additionally attend the clinic for face-to-face review so that a TBSE can be performed.

### Limitations

A limitation of this retrospective study is that, although clinic proformas were documented contemporaneously by clinicians, eliminating recall bias, 555 patients (9.3%) were not included in the study because they did not have documentation of whether TBSE was performed. Explanations included that TBSE was not offered to patients, was declined by patients and not documented, or was performed but not documented. In addition, because this was a retrospective study, groups were not age- or sex-matched at baseline and we found more men and older patients in group A.

The purpose of this cohort study was to determine the detection rates of incidental skin cancers, and we did not set out to assess skin cancer outcomes. There are implications for our findings given that our data noted that most incidental skin cancers detected were BCCs. Nevertheless, BCCs confer sizeable health economic burden^39^ and can lead to significant morbidity in patients, with delays in treatment being associated with further increased health care costs.^40^ A significant proportion of incidental BCCs found in this study (45.7%) were located on high-risk sites, such as the face and neck, and, although we did not assess patient-reported outcomes, it is well recognized that facial skin cancers and their treatment may influence psychological morbidity.^41^ Prospective longitudinal studies are required to determine the impact of TBSE in this setting associated with morbidity, mortality, and health-related costs. Future work should also evaluate the attitudes of clinicians and patients using qualitative approaches to determine the reasons TBSE is not completed or is declined.

## Conclusions

This study appears to support the use of TBSE for patients presenting to urgent skin cancer screening clinics, because a significant proportion of malignant lesions were detected this way and TBSE gave a higher yield of detection compared with population screening. However, most cancers detected incidentally were BCCs, and prospective studies are needed to evaluate the health economic impact of TBSE. The likelihood of detecting an incidental malignant lesion was significantly higher in individuals who presented with a clinically suspicious index lesion warranting biopsy, compared with those who presented with a clinically benign index lesion. These findings could have potential implications for the widespread increased use of teledermatology in the skin cancer management pathway where TBSE is largely excluded.

## Notes

Supplement. **eTable 1.** Age Distribution of Patients With Melanomas **eTable 2.** Index and Incidental Invasive Melanoma Breslow Thicknesses **eFigure.** Distribution of Malignant Incidental Lesions According to Sex
