# Supplementary material for: Identification of Incidental Skin Cancers Among Adults Referred to Dermatologists for Suspicious Skin Lesions
Source: JAMA Netw Open. 2020 Dec 16;3(12):e2030107. doi: 10.1001/jamanetworkopen.2020.30107 (PMC7745102; doi:10.1001/jamanetworkopen.2020.30107)
Supplement: Supplement. — eTable 1. Age Distribution of Patients With Melanomas eTable 2. Index and Incidental Invasive Melanoma Breslow Thicknesses eFigure. Distribution of Malignant Incidental Lesions According to Sex [file jamanetwopen-e2030107-s001.pdf]

Supplementary Online Content

Omara S, Wen D, Ng B, et al. Identification of incidental skin cancers among adults referred to dermatologists for suspicious skin lesions. *JAMA Netw Open*. 2020;3(12):e2030107. doi:10.1001/jamanetworkopen.2020.30107

eTable 1. Age Distribution of Patients With Melanomas

eTable 2. Index and Incidental Invasive Melanoma Breslow Thicknesses

eFigure. Distribution of Malignant Incidental Lesions According to Sex

This supplementary material has been provided by the authors to give readers additional information about their work.

© 2020 Omara S et al. *JAMA Network Open.*

eTable 1: Age distribution of patients with melanomas

Age range	Patients with index melanomas	Patients with incidental melanomas	Group A incidental melanomas	Group B incidental melanomas
30 and under	7	0	0	0
31-40	11	1	1	0
41-50	27	2	1	1
51-60	40	3	3	0
61-70	41	2	1	1
71-80	37	6	5	1
81-90	22	8	5	3
91-100	5	1	1	0
Total	190	23	17	6

© 2020 Omara S et al. *JAMA Network Open.*

eTable 2: Index and incidental invasive melanoma Breslow thicknesses

Invasive melanoma Breslow thickness (mm)	Index lesions	Incidental lesions
**Mean (SD)**	1.99 (2.57)	0.71 (0.38)
**Median (IQR)**	0.95 (1.87)	0.73 (0.56)
**Range**	16.6	1.12

© 2020 Omara S et al. *JAMA Network Open.*

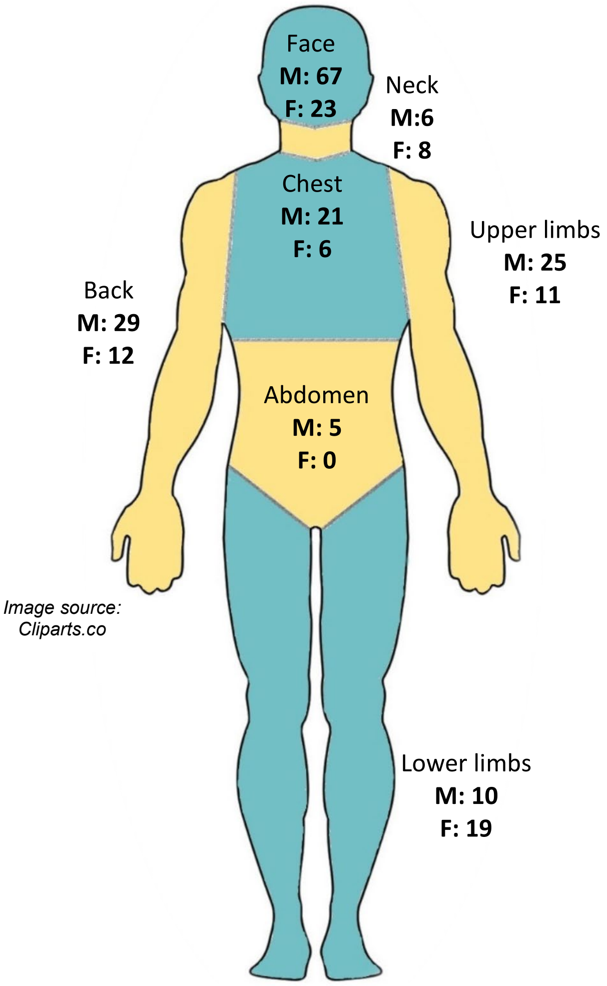

eFigure. Distribution of malignant incidental lesions according to sex

© 2020 Omara S et al. *JAMA Network Open.*
